# Conservative Treatment in Diverticulitis Patients with Pericolic Extraluminal Air and the Role of Antibiotic Treatment

**DOI:** 10.1007/s11605-019-04153-9

**Published:** 2019-03-11

**Authors:** H. E. Bolkenstein, S. T. van Dijk, E. C. J. Consten, B. G. F. Heggelman, C. M. A. Hoeks, I. A. M. J. Broeders, M. A. Boermeester, W. A. Draaisma

**Affiliations:** 1 University of Twente, 5, Drienerlolaan, 7522 NB Enschede, The Netherlands; 2 Department of Surgery, Meander Medisch Centrum, 3800 BM Amersfoort, The Netherlands; 3 Department of Surgery, Academic Medical Centre, Amsterdam, The Netherlands; 4 Department of Radiology, Meander Medical Centre, Amersfoort, The Netherlands; 5 Department of Surgery, Meander Medical Centre, Amersfoort, The Netherlands; 6 Department of Surgery, Jeroen Bosch Hospital, ‘s-Hertogenbosch, The Netherlands

**Keywords:** Diverticulitis, Pericolic air, Conservative treatment, Antibiotic treatment

## Abstract

**Background:**

Recently published studies advocate a conservative approach with observation and antibiotic treatment in diverticulitis patients with pericolic air on computed tomography (CT). The primary aim of this study was to assess the clinical course of initially conservatively treated diverticulitis patients with isolated pericolic air and to identify risk factors for conservative treatment failure. The secondary aim was to assess the outcome of non-antibiotic treatment.

**Methods:**

Patient data from a retrospective cohort study on risk factors for complicated diverticulitis were combined with data from the DIABOLO trial, a randomised controlled trial comparing non-antibiotic with antibiotic treatment in patients with uncomplicated diverticulitis. The present study identified all patients with Hinchey 1A diverticulitis with isolated pericolic air on CT. Pericolic air was defined as air located < 5 cm from the affected segment of colon. The primary outcome was failure of conservative management which was defined as need for percutaneous abscess drainage or emergency surgery within 30 days after presentation. A multivariable logistic regression of clinical, radiological and laboratorial parameters with respect to treatment failure was performed.

**Results:**

A total of 109 patients were included in the study. Fifty-two (48%) patients were treated with antibiotics. Nine (8%) patients failed conservative management, seven (13%) in the antibiotic treatment group and two (4%) in the non-antibiotic group (*p* = 0.083). Only (increased) CRP level at presentation was an independent predictor for treatment failure.

**Conclusions:**

Conservative treatment in diverticulitis patients with isolated pericolic air is a suitable treatment strategy. Moreover, non-antibiotic treatment might be reasonable in selected patients.

## Introduction

Colonic diverticulosis is primarily seen in the Western population with prevalence increasing with age. At 40 years of age, approximately 10% of the Western population has diverticulosis, while this number increases up to 70% in octogenarians. About 4–7% of patients with diverticulosis will develop diverticulitis.^1,2^ Twenty-five per cent of diverticulitis patients develops complications such as abscess or colonic perforation.^3^ Uncomplicated diverticulitis is usually treated conservatively, whereas complicated diverticulitis is treated with percutaneous abscess drainage or operative intervention (emergency resection or laparoscopic lavage).^4^ Most classification systems that categorise diverticulitis are based on computed tomography (CT) findings, such as fluid collections, extraluminal contrast leakage or free air.^5–8^ The clinical relevance of free air on CT remains unclear. Large amounts of distant free air on CT is usually considered a sign of diffuse peritonitis and warrants caution as operative intervention is often needed, whereas patients who present with isolated pericolic air may be treated conservatively.^9^ Current guidelines on treatment of diverticulitis do not describe uncomplicated diverticulitis patients with isolated pericolic air nor do they advise on the optimal treatment strategy.^10^ Recently published studies advocate a conservative approach with observation and antibiotic treatment. These studies are however hampered by small or heterogeneous study populations (including patients with pericolic and distant free air) or inadequate outcome parameters (not including percutaneous drainage as a treatment failure), hindering direct translation to clinical practice.^11–15^ The primary aim of the present study was therefore to assess the course of uncomplicated diverticulitis patients with isolated pericolic air seen on CT imaging and to identify risk factors for failure of conservative treatment. The second aim was to assess the outcome of non-antibiotic treatment in this patient group.

## Materials and Methods

### Study Design

The present study was a joint venture between the DIABOLO trial^16^, a multicenter randomised controlled trial comparing antibiotic with non-antibiotic treatment in 528 patients with uncomplicated acute diverticulitis^17–19^, and a retrospective cohort study in 943 patients studying risk factors for complicated diverticulitis performed in the Meander Medical Centre. The DIABOLO^16^ trial prospectively included all patients with Hinchey 1A and 1B diverticulitis between June 2010 and October 2012 whereas for the retrospective cohort, a diagnostic specific code was used to identify all patients presenting with an episode of diverticulitis in the emergency department between January 2005 and January 2017. The study was approved by the local Institutional Review Board of the Meander Medical Centre.

### Study Population

This study included all patients with an uncomplicated diverticulitis (only modified Hinchey 1A)^17–19^ with pericolic air on CT. Only patients presenting without signs of sepsis and no clinical or radiological evidence of an abscess or diffuse peritonitis at presentation were included.^20^ Patients who received emergency surgery or abscess drainage within 24 h after presentation were also excluded. Imaging was performed using spiral CT scanners (Siemens SOMATOM: Sensation 16, Definition AS, Definition Flash) with the patient in supine position. Axial slices were spaced at 3 mm (mm) (Definition AS / Flash) or 5 mm (Sensation 16) intervals and contained 512 × 512 pixels. Intravenous contrast (Xenetix 300/350, Guerbet, The Netherlands) was administered (unless the patient had a contraindication for intravenous contrast). All CT-reports were checked for mentioning of the following signs; “pericolic air bubbles or pockets”, “pericolic free air or gas”, “intraperitoneal free air or gas”, “extraluminal air” or “covered perforation”. Subsequently, these CTs were re-analysed by two independent radiologists for the presence and classification of extraluminal air on CT. Both radiologists were blinded for patient characteristics, initial CT report from the participating hospital, CT report from the other expert reader and patient outcome. In line with previous published literature^11–15^, pericolic air was defined as air located less than 5 cm from the affected segment of colon, regardless of whether the air was intra- or retroperitoneal. Only patients in whom both radiologists reported extraluminal air < 5 cm from the affected segment were included. Patients without extraluminal air or extraluminal air > 5 cm from the affected segment were excluded from analysis. The volume of extraluminal air was estimated by measuring the air pocket’s largest diameter in two directions in the axial plane and in the coronal plane. The presence of free fluid was scored, as well as the location of free fluid (pericolic, Douglas’ pouch or diffuse).

### Data Collection and Outcomes

Patient characteristics, clinical signs and symptoms, American Society of Anesthesiologists (ASA) Physical Status classification scores, laboratory parameters (C-reactive protein (CRP) and leucocyte level at presentation), CT-findings and initial treatment strategy (e.g. antibiotic treatment, watchful waiting), were collected from the hospital records. The primary outcome was failure of conservative management which was defined as need for emergency surgery or percutaneous abscess drainage within 30 days after presentation. The occurrence of failure of conservative treatment was determined retrospectively in both study cohorts. Secondary outcome measures were length of hospital stay, complications (colonic obstruction, abscess, perforation) and mortality. Moreover, the primary outcome per initial treatment strategy (antibiotic or non-antibiotic treatment) was assessed. For the patients from the retrospective cohort, patients were assigned to the antibiotic treatment group if antibiotic treatment had been started within 24 h after presentation. Antibiotic treatment was not started according to a predefined protocol but at the discretion of the attending physician.

### Statistical Analysis

Descriptive statistics were provided of all variables. Continuous variables are presented as means with standard deviation (SD) or medians with inter quartile range (IQR) according to their distribution. For categorical variables, counts and percentages are presented. Categorical variables were compared using the chi-square test or Fisher’s exact test, as appropriate, and continuous variables were compared using the independent *t* test or the Mann–Whitney *U* test, as appropriate. Multivariable logistic regression was performed to identify risk factors for failure of conservative treatment. Variables that were univariably associated (*p* < 0.20) with failure of conservative treatment were entered into the multivariable model. Odds ratios are presented with 95% confidence intervals. Two sided *P* < 0.05 was considered statistically significant. All analyses were performed using the statistical software package SPSS 24.0 (IBM Corporation, New York, USA).

## Results

### Patient Characteristics

**Fig. 1:**
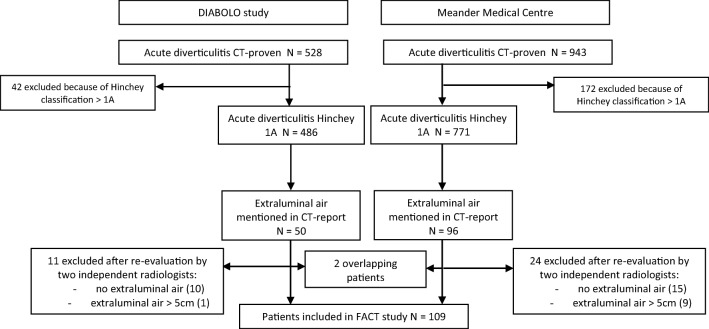
Flowchart of study patients

Figure 1 depicts the selection of patients presenting with acute Hinchey 1A diverticulitis with pericolic air on CT form the two cohorts. In total, 1471 diverticulitis patients were identified. Of these, 214 patients were excluded because of clinical or radiological signs of peritonitis or abscess (Hinchey classification > 1A). In 146 (12%) of the 1257 Hinchey 1A patients, extraluminal air was reported in the initial CT reports. These patients were all initially treated conservatively. Thirty-five patients were excluded because at re-evaluation, no extraluminal air was observed (*n* = 25) or the extraluminal air was located more than 5 cm of the affected segment (*n* = 10). After exclusion of two duplicate patients, a total of 109 patients were included in the present study; 39 patients from the DIABOLO^14^ trial and 70 patients from the retrospective single-centre cohort. Baseline characteristics are shown in Table 1. The mean age was 53 years (SD 11) and 33% of the patients were female. Median amount of extraluminal air was 1.5 cc (IQR 1.0–2.5). Radiologists reported free fluid in 12 (11%) patients which was most frequently seen in Douglas’ pouch (*n* = 11). Fifty-two (48%) patients received antibiotic treatment within 24 h after presentation. Baseline characteristics were mostly comparable between the antibiotic and non-antibiotic group. CRP level seemed to be slightly higher in the antibiotic group (median 142 versus 115 mg/L). The volume of pericolic air was significantly higher in the antibiotic group (median 2.0 cc versus 1.5 cc) compared to the non-antibiotic group.

**Table 1:** Patient characteristics

	All patients (*N* = 109)	No antibiotics (*N* = 57)	Antibiotic treatment (*N* = 52)^a^	*P* value
Patient demographics				
Age, mean (SD)	53 (11)	52 (11)	55 (12)	0.137^c^
Female gender, *N* (%)	36 (33%)	22 (39%)	14 (27%)	0.196^d^
ASA classification				0.403^e^
ASA I	65 (60%)	37 (65%)	28 (54%)	
ASA II	38 (35%)	18 (32%)	20 (38%)	
ASA III	6 (5%)	2 (4%)	4 (8%)	
BMI kg/m^2^, mean (SD)	27.6 (4.6)	26.6 (3.6)	28.9 (5.4)	0.026^c^
History of diverticulitis, *N* (%)	10 (9%)	6 (10%)	4 (9%)	0.496^d^
Clinical status				
Temperature °C, mean (SD)	37.6 (0.8)	37.5 (0.8)	37.7 (0.8)	0.118^c^
Laboratory findings				
CRP (mg/L), median (IQR)^b^	124 (76–199)	115 (73–179)	142 (90–218)	0.106^4^
Leucocytes (10^9/L), mean (SD)^b^	14.8 (4.1)	14.7 (3.6)	14.9 (4.6)	0.803^c^
CT findings				
Volume of pericolic air (CC), median IQR	1.5 (1.0–2.5)	1.5 (1.0–2.0)	2.0 (1.5–3.0)	0.019^f^
Intraperitoneal fluid, *N* (%)	12 (11%)	9 (16%)	3 (6%)	0.096^d^

*SD* standard deviation, *IQR* inter quartile range, *ASA* American Society of Anesthesiologists, *BMI* body mass index, *CRP* C-reactive protein, *WBC* white blood cell

^a^Within 24 h after presentation

^b^At presentation

^c^Independent *T* test

^d^Chi^2^ test

^e^Fisher’s exact test

^f^Mann-Whitney *U* test

### Primary Outcome: Failure of Conservative Management

Table 2 shows the failure of conservative management. Nine of 109 (8%) patients had failure of conservative management, 2 (2%) patients required percutaneous abscess drainage and 7 (6%) patients required emergency surgery within 30 days after presentation. Of the patients who required emergency surgery, a second CT was made in four patients because of clinical deterioration and increasing abdominal pain. These CTs revealed an abscess in two patients and no deterioration of disease in the other two patients. Three of these patients had purulent peritonitis upon surgery, whereas in one patient, no deterioration of disease was seen. Three of the patients who required emergency surgery did not undergo a second CT, but a diagnostic laparoscopy was performed in these patients where a purulent peritonitis was seen.

**Table 2:** Treatment failure and subgroup analysis of DIABOLO patients

Present study	All patients (*N* = 109)	No antibiotics (*N* = 57)	Antibiotic treatment (*N* = 52)^a^	*P* value^b^
Percutaneous drainage *N* (%)	2 (2%)	0 (0%)	2 (4%)	0.23
Emergency surgery *N* (%)	7 (6%)	2 (4%)	5 (10%)	0.26
Total treatment failure *N* (%)	9 (8%)	2 (4%)	7 (13%)	0.08
DIABOLO sub analysis	All patients (*N* = 39)	No antibiotics (*N* = 22)	Antibiotic treatment (*N* = 17)	*P* value^b^
Percutaneous drainage *N* (%)	0 (0%)	0 (0%)	0 (0%)	–
Emergency surgery *N* (%)	1 (3%)	0 (0%)	1 (6%)	0.44
Total treatment failure *N* (%)	1 (3%)	0 (0%)	1 (6%)	0.44

^a^Received antibiotics within 24 h after presentation

^b^Fisher’s exact

One patient underwent open sigmoidectomy with diverting ileostomy, three patients underwent laparoscopic sigmoid resection with primary anastomosis, two patients underwent Hartmann procedure and one patient received laparoscopic lavage. Overall median time to treatment failure was 3 (IQR 2–5) days.

### Outcome per Initial Treatment Strategy

Table 2 shows the failure of conservative management per initial treatment strategy, as well as a subgroup analysis of the DIABOLO^14^ patients (who were randomly assigned to either antibiotic or non-antibiotic treatment). Seven of 52 (13%) patients in the antibiotic treatment group failed conservative treatment versus 2 of 57 (4%) patients in the non-antibiotic group. There was no statistically significant difference in failure of conservative treatment between patients treated with and without antibiotics (*p* = 0.083). In a subgroup analysis of the 39 DIABOLO^14^ patients, only one patient failed conservative treatment in the antibiotic group versus nil in the non-antibiotic group (*p* = 0.44).

### Secondary Outcome: Mortality, Complications, Re(Admittance) and Hospital Stay

One patient died due to persistent abdominal sepsis following a Hartmann’s procedure. Eleven (10% [11/109]) patients developed complications; colonic obstruction (*n* = 1), perforation (*n* = 5) and abscess (*n* = 5). Forty (37%) [40/109]) patients were treated as outpatients of which two were eventually admitted to the hospital due to clinical deterioration or development of complications. Fourteen (35% [14/40]) of the outpatients were treated with antibiotics. Sixty-nine (63% [69/109]) patients were treated as inpatients of which seven (10% [7/69]) patients were re-admitted to the hospital within 30 days after presentation due to clinical deterioration or development of complications. Thirty-eight (55% [38/69]) of the inpatients were treated with antibiotics. Two (4%) patients in the non-antibiotic treatment group were started on antibiotics more than 24 h after presentation due to clinical deterioration or development of complications. Median length of hospital stay was 3 days (IQR 2–5).

### Long-Term Outcomes

Median follow-up was 11 months (IQR 2–24). Twenty-five of 109 (23%) patients developed recurring diverticulitis, all of whom were treated conservatively. Of the patients with treatment failure (*n* = 9), one (11%) patient developed a recurrence. Nineteen of 109 (17%) patients underwent elective sigmoidectomy. Indications for these resections were stenosis (*n* = 3), fistula (*n* = 3) and recurring diverticulitis or persistent complaints (*n* = 13). One patient died due to non-diverticulitis related disease.

### Risk Factors for Treatment Failure

Table 3 shows the univariable and multivariable analyses of factors associated with failure of conservative management (need for percutaneous abscess drainage or emergency surgery). Location of free fluid was not included in the analysis as one radiologist scored these all in Douglas’ pouch. The initial treatment strategy (non-antibiotic or antibiotic treatment) was included in the multivariable analysis to correct for a possible treatment effect of antibiotics. In the multivariable analysis, only CRP level (OR 1.01 for each mg/L increase; 95% CI 1.001–1.02) remained statistically significant. Although not statistically significant in the multivariable analysis, leucocyte count and age seemed to be higher in the treatment failure group (mean 18.2 × 10^9/L vs 14.5 × 10^9/L and mean 60 vs 52 years, respectively).

**Table 3:** Factors associated with failure of conservative management

	Treatment success (*N* = 100)	Treatment failure (*N* = 9)	*P* value	Unadjusted odds ratio (95%CI)^a^	Adjusted odds ratio (95%CI)^b^
Patient demographics					
Age, mean (SD)	52 (10)	60 (13)	0.063^e^	1.06 (0.996–1.13)	1.03 (0.96–1.10)
Female gender	34 (34%)	2 (22%)	0.715^g^	1.80 (0.36–9.16)	–
ASA classification					
			0.837^g^		–
ASA I	60 (60%)	5 (56%)		Reference 1.41 (0.36–5.61) –^c^	
ASA II	34 (34%)	4 (44%)			
ASA III	6 (6%)	0 (0%)			
BMI kg/m^2^, mean (SD)	27.7 (4.6)	25.6 (3.9)	0.369^e^	0.86 (0.63–1.18)	–
History of diverticulitis	9 (10%)	1 (11%)	1.000^g^	0.87 (0.10–7.96)	–
Clinical status					
Temperature °C, mean (SD)	37.6 (0.8)	37.5 (0.9)	0.723^e^	0.85 (0.35–2.07)	–
Laboratory findings					
CRP (mg/L), median (IQR)	124 (75–192)	218 (97–364)	0.029^4^	1.01 (1.004–1.02)	1.01 (1.001–1.02)
Leucocytes (10^9/L), mean (SD)	14.5 (4.0)	18.2 (3.6)	0.008^e^	1.24 (1.05–1.48)	1.20 (0.99–1.45)
CT findings					
Volume of pericolic air (CC), median IQR	1.5 (1.0–2.5)	2.0 (1.0–4.0)	0.806^h^	1.02 (0.98–1.06)	–
Intraperitoneal fluid	11 (11%)	1 (11%)	1.000^g^	0.94 (0.11–8.28)	–
Initial treatment					
Antibiotic treatment^d^	45 (45%)	7 (78%)	0.083^f^	4.28 (0.85–21.62)	2.48 (0.43–14.40)

*SD* standard deviation, *IQR* inter quartile range, *NS* not selected, *CRP* C-reactive protein, *ASA* American Society of Anesthesiologists, *BMI* body mass index, *CI* confidence interval

^a^Odds ratio in univariable analysis

^b^Odds ratio in multivariable analysis

^c^Odds ratio could not be calculated because zero events occurred in this group

^d^Within 24 h after presentation

^e^Independent *T* test

^f^Chi^2^ test

^g^Fisher’s exact test

^h^Mann-Whitney *U* test

## Discussion

The present study analysed the course of diverticulitis patients presenting with isolated pericolic air on CT. The vast majority (92%) of patients recovered with conservative treatment. This indicates that diverticulitis patients with isolated pericolic air on CT can safely be treated conservatively.

The clinical relevance of pericolic air on CT in diverticulitis patients presenting without signs of generalised peritonitis or sepsis has been topic of debate. Several studies have recently been published reporting on the non-operative management of perforated diverticulitis. Titos-Garcia et al.^14^ and Salinnen et al.^12^ specifically report non-operative treatment success rates for patients with isolated pericolic air, 90 and 99% respectively. Both studies only considered emergency surgery as treatment failure. These findings are consistent with our finding that 92% of the patients with isolated pericolic air recovered with conservative treatment.

Most studies reporting on the non-operative management of perforated diverticulitis include patients with pericolic and distant free air. The present study only included patients with isolated pericolic air. We selected this patient group because literature suggests that pericolic and distant extraluminal air have a different disease course and demand different treatment strategies. In a patient with distant free air, we can no longer speak of a “covered perforation” as the perforation is no longer contained to the pericolic region. This is why large amounts of free distant air is usually considered a sign of diffuse peritonitis and an indication for operative intervention whereas patients with isolated pericolic air may be treated conservatively. Recent studies report contrastingly about the risk of conservative treatment failure in patient with distant free air. Sallinen et al.^12^, Colas et al.^15^ and Titos-Garcia et al.^14^ report lower success rates of conservative treatment in patients with distant free air; 62%, 59% and 62%, respectively. Contrastingly, Dharmarajan et al.^13^ and Costi et al.^11^ report no difference in outcome between pericolic and distant free air. However, the number of patients presenting with distant free air was relatively small in these studies, hampering proper comparison. As patients with free distant air do show a tendency towards a more complicated course, including these patients would have led to a heterogeneous study population. We therefore chose to only include patients with isolated pericolic air to assess the outcome of (non-antibiotic) conservative treatment.

The question remains which therapeutic approach we should adopt in diverticulitis patients with pericolic air on CT. A recent systematic review, including the studies mentioned above, concludes that conservative treatment in patients with pericolic air is justifiable but should include antibiotic treatment, as all patients included in this study received intravenous antibiotics as part of their non-operative management.^21^ Since we found a conservative treatment success rate of 92% in the present study, we agree that conservative treatment is appropriate in this patient group. The rationale for antibiotic treatment is however not well-founded and might be questioned. Fifty-seven (52%) of our patients were treated without antibiotics and of these, 55 (96%) patients were treated successfully. The baseline characteristics of the antibiotic and non-antibiotic group were mostly comparable. C-reactive protein (CRP) seemed to be slightly higher in the antibiotic group (median 142 versus 115 mg/L), and the volume of pericolic air was significantly higher in the antibiotic group (median 2.0 versus 1.5 cc) compared to the non-antibiotic group. The clinical relevance of this marginal difference is however debatable, especially since accurate measurement of amount of free air on CT is difficult and subjective. We found no statistical significant difference in failure of conservative treatment between patients treated with and without antibiotics (*p* = 0.083). This could indicate that antibiotic treatment might not be mandatory in patients with pericolic air. However, 64% of the study population came from a retrospective database and in these patients, antibiotic treatment was started at the discretion of the attending physician. Therefore, there is a high risk of confounding by indication. It is possible that patients who presented with more severe illness (who might be at higher risk of conservative treatment failure) were more likely to receive antibiotic treatment and therefore, these findings should be interpreted very carefully. In a subgroup analysis of the DIABOLO^14^ patients (who were randomly assigned to either watchful waiting or antibiotic treatment), there was also no difference in outcome found between patients treated with and without antibiotics, strengthening our conclusion that antibiotics may not be mandatory in patients with pericolic air. However, further research aimed at the non-antibiotic treatment of diverticulitis patients with isolated pericolic air should be performed before a conservative treatment strategy without antibiotics can safely be assumed. Sixty-three [69/100] percent of the patients were directly admitted to the hospital, whereas 37% [40/109] were treated as outpatients. The decision to admit a patient to the hospital was made by the attending physician based on individual patient characteristics. We chose to include both inpatients and outpatients in our analysis as previous literature has provided strong evidence that in-hospital treatment of patients with uncomplicated diverticulitis does not have a beneficial effect compared to outpatient treatment.^22–27^

A major strength of this study is its multicenter design and the fact that one third of the study population came from a prospective, randomised study. Contrastingly, the other two-third of the study population came from a retrospective database which comes with inherent limitations. Since we were dependent on the information that was recorded in the patient files of the retrospective cohort, we could not analyse all potential risk factors for treatment failure such as body mass index or immunosuppressive medication as we had too little data on these factors. The fact that two independent radiologist re-analysed all CTs enhances the validity of our results. However, a limitation is that we did not re-analyse all CT scans in which free air was not mentioned in the CT-report, to confirm the absence of free air. It could therefore be that we missed a few patients with free air. Moreover, in the DIABOLO study, diverticulitis-positive findings led to CT within 24 h and it could be that these patients had resolution of their free air in that meantime.

The present study is limited by the small number of patients with treatment failure. In the multivariable analysis, only a higher CRP level remained as a significant predictor of treatment failure. Although not statistically significant, a higher leucocyte count and higher age seemed to be associated with treatment failure. Because of the small number of treatment failures, statistical power might have been insufficient to identify risk factors for treatment failure. The primary aim of this study was however to assess the feasibility of (non-antibiotic) conservative management in patients with pericolic air, and the small number reflects the low probability of treatment failure in patients with isolated pericolic air.

## Conclusion

Conservative management in patients with acute diverticulitis with isolated pericolic air is a suitable treatment strategy. It however remains uncertain whether antibiotic treatment is necessary in patients with isolated pericolic air, due to the low event rate. A higher CRP level was significantly associated with treatment failure, and a higher leucocyte count and higher age showed a non-significant trend towards an association with treatment failure. Patients with isolated pericolic air who present with these risk factors may not be suitable for a conservative treatment strategy and need close observation and/or treatment with antibiotics.
